# Effects of Physical Exercise Programs on Sarcopenia Management, Dynapenia, and Physical Performance in the Elderly: A Systematic Review of Randomized Clinical Trials

**DOI:** 10.1155/2019/1959486

**Published:** 2019-11-20

**Authors:** Renato Gorga Bandeira de Mello, Roberta Rigo Dalla Corte, Joana Gioscia, Emilio Hideyuki Moriguchi

**Affiliations:** ^1^School of Medicine at the Federal University of Rio Grande do Sul (UFRGS), Department of Internal Medicine, Porto Alegre, Brazil; ^2^Hospital de Clínicas de Porto Alegre, Section of Internal Medicine, Porto Alegre, Brazil; ^3^Post-Graduation Studies Program in Endocrinology, UFRGS, Porto Alegre, Brazil; ^4^School of Medicine at the Federal University of Rio Grande do Sul (UFRGS), Porto Alegre, Brazil; ^5^Post-Graduation Studies Program in Cardiology and Cardiovascular Sciences, UFRGS, Porto Alegre, Brazil

## Abstract

**Introduction:**

Sarcopenia is a prevalent condition in the elderly population, imposing a significant impact over their functional ability as well as their quality of life. Furthermore, it is associated with greater incidence of major geriatric outcomes, as reduced mobility, falls, loss of independence, cognitive impairment, and all-cause mortality. Physical Exercise Programs directed to improve muscle mass and its function may be key to reduce sarcopenia consequences. However, a significant heterogeneity is found in clinical trials, especially as a consequence of different exercise protocols applied to research subjects.

**Objectives:**

To access the effects of physical exercise programs compared to no exercise interventions to improve sarcopenia components and its determinants in sarcopenic elder individuals.

**Methods:**

A systematic review was conducted in the Pubmed database to identify randomized clinical trials (RCTs) which tested the effects of physical exercise programs to manage sarcopenia components in sarcopenic elder individuals. Two independent reviewers assessed the studies' eligibility according to specified inclusion criteria in a four-step strategy. Data regarding population characteristics, muscle mass, muscle quality, muscle strength, and muscle function were extracted from each one of the included studies. Assessment of quality and individual studies risk of bias were assessed through Cochrane Risk of Bias Tool®. Assuming theoretical expected heterogeneity among studies, especially regarding different physical exercise programs and different outcome measurements, authors decided to be conservative and present study results in descriptive tables.

**Results:**

Search strategy retrieved 298 papers on PubMed database. Three more were identified through manual search, being 301 studies revised for inclusion. 278 were excluded during title/abstract review. After further evaluation of 23 full-texts, 5 RCTs were included. All 5 trials tested the efficacy of isolated exercise programs to improve sarcopenia components in the elderly compared to no physical intervention. Resistance training was the main intervention component in all included trials compared to inactive control groups (health education mainly). Physical training improved muscle strength, muscle quality, and muscle function compared to inactive control groups. Considering muscle mass, no differences were demonstrated. Data meta-analysis was not possible to be performed due to high heterogeneity among trials and small number of studies for each outcome comparison.

**Conclusion:**

Heterogeneity among trials and small number of RCTs limited robust conclusions and data meta-analysis. However, resistance training protocols can improve muscle strength and physical performance in elders previously diagnosed with sarcopenia, although its effect size and clinical impact are barely relevant.

## 1. Introduction

According to the European Working Group on Sarcopenia in older people revised consensus, sarcopenia is a skeletal muscle disorder in which muscle strength is the key feature of a clinical condition with increased risk for major geriatric outcomes [1]. It is a prevalent condition in the elderly, varying according to age-related variables especially when different clinical settings where compared. In community-dwelling samples, a wide prevalence range was found from 1% to 29%; and in long-term care facilities, the range is 14–33% [2]. However, it is presumed that this heterogeneity would be also explained by different applied diagnostic criteria.

It is postulated that physical exercise programs can shift sarcopenia clinical course. A systematic review was conducted in 2014, and the authors concluded that physical exercise has an impact on improving muscle strength and physical performance; however, interventions did not significantly improved muscle mass. Several limitations were pointed out to explain the low impact of exercise interventions: lack of standardization of exercise protocols, low duration of interventions, heterogeneity in outcome measurements, and selection bias due to heterogeneous eligibility criteria.

Most recently, in 2017, the last published systematic review regarding exercise and sarcopenia showed better physical performance after resistance training exercise intervention, but no improvement in muscle strength [3]. Beyond physical exercise impact on physical performance and muscle strength and mass, it is important to access its effects on reducing major geriatric outcomes. Guerreiro et al. demonstrated that both muscle mass estimated by bedside ultrasound and muscle performance and strength in hospitalized elderly patients are important predictors for functional decline, rehospitalization, and death [4]. However, most clinical trials testing physical exercise in sarcopenic elder patients yet do not access its effects over major clinical geriatric outcomes.

Considering the aforementioned reasons, the main objective of this systematic review is to analyze the effectiveness of physical exercise on improving sarcopenia in older populations. Muscle mass, muscle function, muscle strength, and physical resistance improvement in the elderly will be investigated. Furthermore, we will show these effects on the incidence of major geriatric outcomes.

### 1.1. Methods

This systematic review protocol followed the Preferred Reporting Items for Systematic Reviews and Meta-Analyses (PRISMA) recommendations [5].

### 1.2. Design: Systematic Review of Randomized Clinical Trials (RCTs)

#### 1.2.1. Eligibility Criteria

RCT testing effects of physical exercise programs were compared to those of a physically inactive control group on sarcopenia clinical variables in elderly populations previously diagnosed with sarcopenia. The eligible criteria are predefined by the characteristics of the primary studies. At this point, it is only necessary to define the following criteria: the target population, the intervention, and the outcomes. Characteristics of the included study populations, such as intervention types and outcome measure, are presented in Table 1.

Population: elderly (>65 years) with diagnosed sarcopenia; intervention and control: physical exercise programs compared to a control group (no exercise); outcomes: sarcopenia, muscle mass, muscle strength, physical performance, and muscle quality; length of follow-up: not specified; study design: randomized clinical trial. There was no limitation of gender. The types of exercises were predominantly resistance training (Table 1).

#### 1.2.2. Search Strategy

A systematic search was conducted in the PubMed electronic articles database using the following strategy: (((Sarcopenia) AND (Elderly)) AND ((Physical activity) OR (Exercise)) AND (Clinical trial)). No specific date limit was defined; no language limitation was imposed; all available studies were included. Last search was conducted on June 30th, 2019. For those articles with limited access or incomplete data, the authors were contacted directly by email. Additional manual search was performed to increase search sensitivity.

**Figure 1:**
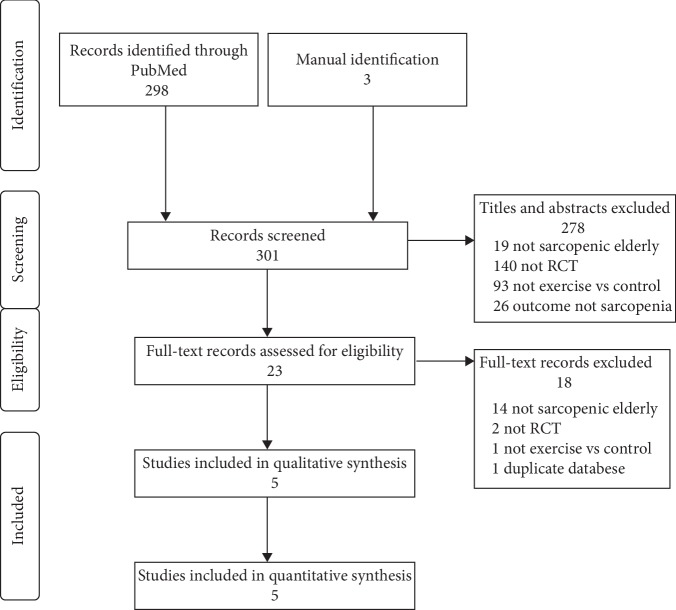
Flowchart of records retrieved, screened, and included.

*(1) Study Selection and Data Collection Process*. Step 1: two independent reviewers assessed all titles and abstracts to verify eligibility criteria on Revision. Step 2 included a full-text revision for further eligibility assessment. Step 3: duplicates were excluded. Final inclusion results are presented in the systematic review inclusion flowchart (Figure 1). A standardized Microsoft Office ExcelTM spreadsheet was used to organize independent data collection. Investigators followed a step by step extraction process according to the PICOTS prespecified strategy, extracting study ´s population data, followed by intervention description, outcomes variables collection, and its main results.

 Quality and individual studies risk of bias were assessed through Cochrane Risk of Bias Tool® [11] and are presented in Table 1.

## 2. Data Analysis

Assuming theoretical expected heterogeneity among studies, especially regarding different physical exercise programs and different outcome measurements, authors decided to be conservative and present study results in descriptive tables. We assumed that lack of studies' exercise protocols standardization as well as lack of outcomes measurement standardization limits data meta-analysis as theoretical homogeneity assumption is not reached.

Publication bias was also assessed by trim and fill strategy. Analyses were performed using the software Comprehensive Meta-AnalysisTM version 3—free trial [12].

### 2.1. Results

A total number of 298 studies were retrieved by search strategy application on PubMed database. Three more studies were found in a previous meta-analysis and included in the next step [3]. 278 studies were excluded in the Step 1 reviewing process. In Step 2, 23 full-text articles were reviewed for further eligibility evaluation and 18 were excluded. The reasons for the exclusion of these studies are described in the flowchart of Figure 1. Finally, 5 randomized clinical trials were included in this present systematic review as described in the inclusion flowchart (Figure 1).

Table 1 presents study details regarding population, study design, interventions, control groups, outcome measurements, and main results [6–10].

All five studies have high to moderate risk of bias according to Cochrane's risk of bias tool. In three studies conducted by Kim et al., direct comparison of physical training against inactive control is only possible in a study subsample composed by two different intervention groups (exercise versus health education groups).

Different measurement protocols were applied to assess outcomes among studies. In two studies—Strasser et al. [6] and Liao et al. [7]—dual-energy X-ray absorptiometry was used to measure muscle mass as well as muscle quality. All studies conducted by Kim et al. [8–10] measured these variables using bioelectrical impedance analysis.

### 2.2. Main Results

#### 2.2.1. Muscle Mass, Muscle Quality, Strength, and Function

Results regarding muscle mass, muscle strength, and muscle quality are summarized in Table 2. Muscle mass was only significantly improved in the RCT conducted by Liao et al. [7] Sarcopenic elder patients submitted to 12 weeks intervention of resistance training (RT) gained almost 1 kg of appendicular muscle mass (AMM) compared to the control group. All other 4 studies did not show muscle mass differences compared to control. However, when muscle quality was analyzed, significant results were found by both Strasser et al. [6] and Liao et al. [7]. Kim et al. did not access the muscle quality in neither 3 studies. Muscle strength was not improved after RT intervention.

RT exercise protocols significantly improved the muscle function measured by gait speed (GS) as well as by the Timed Up and Go test (TUG) in 3 of 5 studies as described in Table 3. Kim et al. [8, 9] did not evidence the muscle function improvement after RT.

In [7], quality of life was accessed before and after exercise interventions and it was possible to show significant improvement in QoL in the RT group when compared to control (mean difference 13.62 (6.47, 20.76); *P* < 0.001), especially a relevant difference in the physical component of the SF-36 questionnaire.

## 3. Discussion

In this systematic review to assess the effectiveness of exercise training to improve sarcopenia-related outcomes in sarcopenic elder populations, a sensitive search strategy retrieved 301 studies on PubMed database. During the first step review process, 278 papers were excluded and 23 more were excluded after full-paper review, leading to 5 RCTs to be included in this study.

RCTs results according to sarcopenia component varied significantly. Only one study evidenced muscle mass gain; muscle quality, on the other hand, was improved in both studies that this factor was measured. Although effects over muscle strength and muscle mass were not clear, muscle function—walking speed and Timed Up and Go test—was homogeneously improved among studies, but the size effect seems to be limited.

However, it is presumed that resistance training prevents muscle mass wasting because it stimulates muscle hypertrophy and increases muscle strength, as postulated by Johnston et al. [13], and also it is postulated that resistance training is a key strategy to treat sarcopenia; only one clinical trial [7] evidenced improvement of muscle mass after a physical exercise protocol was applied in elder individuals previously diagnosed with sarcopenia. One possible explanation resides in lack of power to detect significant differences in the other 4 trials, as sample sizes are quite small. Another reason is the duration of resistance training protocols, especially exercise *volume of training*—defined as the total work sets per exercise session. Peterson et al. demonstrated that the greater the volume training the greater the muscle mass gain [14]. Furthermore, they showed a significant effect attenuation of physical interventions according to aging, one possible strong explanation for lack of exercise training effect.

In comparison with previous systematic reviews that evaluated the effect of physical exercises over sarcopenia components, published in 2014 [2] and 2017 [3], this present review included only RCTs in which physical training protocols alone were compared to control groups to improve muscle associated outcomes in previously diagnosed sarcopenic elderly. Cruz-Jentoft et al. included trials testing the aforementioned interventions in different clinical scenarios, as in frail participants, community-dwelling elderly, and in postoperative hip-replacement therapy patients. Regardless of methodological differences between Cruz-Jentoft and this review, results are similar, i.e., no robust effects were demonstrated in most included RCTs. The present search strategy has resemblance to those used in Yoshimura et al.'s systematic review [3]. Although they tried to meta-analyze data to show summary effects for several dependent variables, most forest plots are provided in less than 3 studies, in discordance with meta-analysis guides recommendations. Theoretically, only 2 studies are needed to perform a meta-analysis, but it may carry several important biases as well as statistical inferences especially when random effect models are chosen; the number of studies matters, according to Guolo and Varin [15]. Besides that, it is possible to point a significant difference in the Yoshimura et al. study: two more studies were added to the state of the art regarding physical activity to manage sarcopenia in the elderly—Strasser et al. [6] and Liao et al. [7] Both studies have more robust methodology than those already included in the Yoshimura review. Its results were also more consistent, showing significant improvement in both muscle mass and muscle quality. Furthermore, these both recent RCTs evidenced improvements in muscle function in sarcopenic elderly submitted to a resistance training protocol, allowing to hypothesize that exercises may have relevant impact over major geriatric outcomes as falls, immobility, and dependence. Moreover, Liao et al. showed better results in quality of life scores in those randomized to physical exercise.

The authors decided not to run data meta-analysis to identify a single summary effect for each dependent variable as a significant heterogeneity among studies was assumed, especially regarding intervention protocols and measurement of sarcopenia components. Also noteworthy is the small sample sizes included in the clinical trials, imputing worrisome power limitations to detect significant outcome differences. All 5 studies have moderate to high risk of bias in accordance with the Cochrane risk of bias tool. Assuming these aforementioned limitations in conjunction with small number of available RCTs, it is not recommended to run data meta-analysis due to high risk of bias as meta-analysis will directly reflect the study biases. Additionally, as described by Borenstein et al. [16], it is very important to avoid “*mixing apples and oranges,*” referring to misplaced comparisons by data meta-analysis from theoretically heterogeneous studies, the specific case found in this systematic review.

## 4. Conclusions

Heterogeneity among trials and small number of RCTs limited robust conclusions and data meta-analysis. However, resistance training protocols can improve muscle strength and physical performance in elders previously diagnosed with sarcopenia, although its effect size and clinical impact are barely relevant. Two trials were published since last available systematic review, both of it showing positive results of resistance training protocols over muscle quality and muscle function as well as better results in quality of life scores.

## Figures and Tables

**Table 1:** Basic characteristics of included randomized controlled clinical trials.

Reference	Population	Design	Intervention	Control	Outcome measurement and definition	Main results
Strasser et al. [6] Moderate RoB^*∗*^	33 women and men (82.4 ± 6.0 years) with impaired health status (mostly sarcopenic)	RCT	Resistance training (RT): 12 weeks elastic band resistance training (*n* = 16)	Control group (CG) (*n* = 17)	Measured by DEXA Skeletal muscle mass: apendicuar lean mass (ALM in kg) Muscle quality: isokinetic force measurement of knee flexion and extension (Nm/kg)	Muscle mass: apendicular lean mass: no significant differences between groups Muscle quality (Nm/kg) Baseline 6 months Extension force RT: 10.1 ± 2.9 12.1 ± 2.6 CG: 11.5 ± 2.5 9.9 ± 3.0 *P*=0.006 Flexion force (MQ) RT: 5.2 ± 1.4 6.8 ± 1.0 CG: 5.7 ± 1.5 5.5 ± 1.5 *P*=0.009

Liao et al. [7] High RoB^*∗*^	56 sarcopenic or obese women (mean ± SD age 67.3 ± 5.1 years)	RCT	Resistance training (RT): 12 weeks of elastic band resistance training (ERT) (*n* = 33)	Control group (CG) matched by age (*n* = 23)	Measured by DEXA Muscle mass—apendicular lean Mass (ALM in kg) Muscle quality (MQ) after lower limb muscle flexion (kg/kg) Physical capacity and function outcomes Timed Up and Go (TUG in s); gait speed (GS in m/s) Quality of life (qol measured by SF-36)	Results presented as mean differences between groups (RT-CG) Muscle mass (kg) ALM: 0.99 (0.33, 1.66) *P* < 0.01 Muscle quality (N/kg) MQ-LE: 1.82 (1.25, 2.39) *P* < 0.01 Function TUG: −1.64 (−2.34, −0.95) *P* < 0.01 GS: 0.14 (0.33, 0.25) *P* < 0.05 QoL SF-36: 13.62 (6.47, 20.76) *P* < 0.001

Kim et al.[8] Moderate RoB^*∗*^	139 sarcopenic elderly women; 69 randomized to resistance training or control group	RCT	Resistance training (RT): 12 weeks elastic band for upper limbs and ankle weight for lower limb training (*n* = 35)	Control group (CG) Health education (*n* = 34)	Measured by bioeletrical impedance analysis (BIA) Apendicular skeletal muscle mass (kg) Performance TUG; GS; grip strength	No differences in muscle mass, strength, and function were observed after intervention

Kim et al.[9] Moderate RoB^*∗*^	138 sarcopenic elderly women; 64 randomized to resistance training or control group	RCT	Resistance training (RT): 12 weeks elastic band for upper limbs and ankle weight for lower limb training. (*n* = 32)	Control group (CG): health education (*n* = 32)	Measured by bioeletrical impedance analysis (BIA) Apendicular skeletal muscle mass (kg) Performance TUG; GS; grip strength	Apendicular muscle mass: no difference Performance Grip strength: no difference GS and TUG: no relevant differences found

Kim et al.[10] Moderate RoB^*∗*^	155 sarcopenic elderly women; 78 randomized to exercise group or control group	RCT	Exercise group (EG): 12 weeks combined training—warm up; strengthening exercise, balance and gait training, and cool down. (*n* = 39)	Control group (CG): health education (*n* = 39)	Measured by bioeletrical impedance analysis (BIA) Apendicular skeletal muscle mass (kg) Performance Walking speed, knee extension strength (Nm/kg)	Apendicular muscle mass: no difference Walking speed (m/s) Baseline 6 months EG: 1.31 ± 0.24 1.50 ± 0.23 *P*=0.007 CG: 1.19 ± 0.21 1.22 ± 0.23 Strength: no difference

RCT = randomized clinical trial; RoB: risk of bias; ^*∗*^in accordance with Cochrane's risk of bias tool.

**Table 2:** Muscle mass, strength, and muscle quality mean differences between groups.

	Muscle mass (kg)					Muscle strength					Muscle quality				
	Baseline		After exercise		Mean difference	Baseline		After exercise		Mean difference	Baseline		After exercise		Mean difference
Study	I	C	I	C		I	C	I	C		I	C	I	C	
Strasser et al. [6]	^*∗*^17.8	^*∗*^17.7	^*∗*^18.3	^*∗*^18.2	I: 0.5 C: 0.5	NA	NA	NA	NA	NA	^&^10.1	^&^11.5	^&^12.1	^&^9.9	I: 2.0

Liao et al. [7]	^*∗∗*^36.5	^*∗∗*^37.0	^*∗∗*^36.8	^*∗∗*^36.5	I: 0.28 C: −0.44	^#^13.6	^#^15.26	^#^21.17	^#^13.59	I: 7.57 C: −1.67	^£^2.47	^£^2.95	^£^4.07	^£^2.49	I: 1.6

Kim et al.[8]	^*∗*^15.79	^*∗*^16.86	^*∗*^13.0	^*∗*^12.9	I: −2.79 C: −3.9 6	181.3	197.5	202.7	204.1	I: 21.4 C: 6.6	NA	NA	NA	NA	NA

Kim et al. [9]	^*∗*^14.79	^*∗*^13.96	^*∗*^14.45	^*∗*^14.11	I: 1.23 C: −0.34	^$^51.39	^$^47.54	^$^49.73	^$^43.13	I: −1.66 C: −4.41	NA	NA	NA	NA	NA

Kim et al. [10]	^*∗*^13.9	^*∗*^13.57	^*∗*^14.19	^*∗*^13.67	I: 0.29 C: 0.1	^&^1.12	^&^1.14	^&^1.14	^&^1.0	I: 0.02 C: −0.14	NA	NA	NA	NA	NA

I: intervention; C: control; NA not available; ^#^N: Newton; ^$^Nm: Newton meter; ^&^Nm/kg: Newton meter/kilogram; ^£^N/kg” Newton/kilogram; ^*∗*^ASM: appendicular skeletal muscle mass; ^*∗∗*^FFM: fat free mass.

**Table 3:** Muscle function mean differences between groups.

	Walking speed					TUG^*∗*^				
	Baseline		After exercise		Mean difference	Baseline		After exercise		Mean difference
Study	I	C	I	C		I	C	I	C	
Liao et al. [7]	1.51	1.16	1.53	1.14	I: 0.02 C: −0.02	8.4	9.51	7.08	9.45	I: 7.57 C: −1.67

Kim et al. [8]	1.1	1.1	1.3	1.2	I: 0.2 C: 0.1	NA	NA	NA	NA	NA

Kim et al. [9]	1.26	1.27	1.36	1.26	I: 0.1 C: −0.01	8.81	8.43	7.03	8.88	I: −1.66 C: −4.41

Kim et al. [10]	1.31	1.19	1.5	1.22	I: 0.19 C: 0.03	NA	NA	NA	NA	NA

I: intervention; C: control; NA: not available; TUG: Timed Up and Go test; ^*∗*^walking speed in m/s (meters/second); TUG in s (seconds).
